# Antibody Therapy for Patients with Lymphoid Malignancies: Past and Present

**DOI:** 10.3390/ijms26041711

**Published:** 2025-02-17

**Authors:** Jacob Naman, Nirja Shah, Benjamin M. Heyman

**Affiliations:** 1 Department of Medicine, UC San Diego Health, La Jolla, CA 92037, USA; jnaman@health.ucsd.edu; 2 UCSD School of Medicine, La Jolla, CA 92037, USA; nis004@health.ucsd.edu; 3 Department of Medicine, Division of Regenerative Medicine, UC San Diego Health, La Jolla, CA 92037, USA

**Keywords:** antibody, malignancy, CLL, FL, DLBCL, rituximab, obinutuzumab, polatuzumab, mosunetuzumab, epcoritamab

## Abstract

Antibody therapies are a crucial component of modern lymphoid malignancy treatment and an exciting area of active research. We performed a review of modern antibody therapies used in the treatment of lymphoid malignancies, with an emphasis on landmark studies and current directions. We describe the indications for rituximab, obinutuzumab, ADCs, and bispecific antibody therapies. Finally, we summarize early data from ongoing trials on emerging novel therapy combination regimens and discuss the role of machine learning in future therapy development.

## 1. Introduction

**Figure 1:**
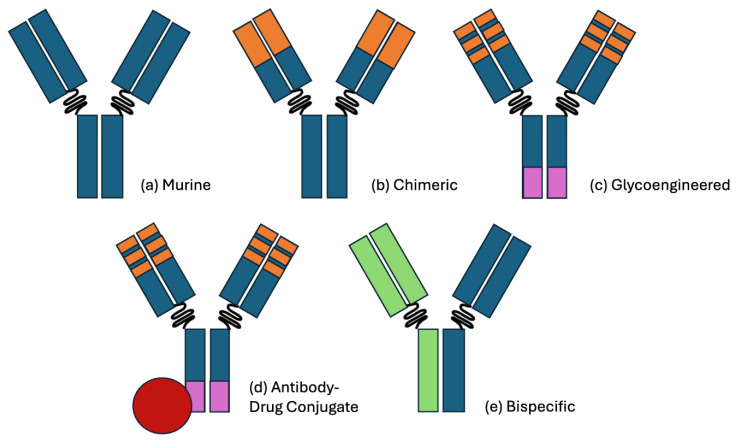
Visual representation of the antibody therapy classes. (**a**) A pure murine antibody, as derived from hybridoma cell lines. (**b**) A chimeric antibody with humanized (orange) fAb regions, improving the immunogenicity and half-life. A notable example is rituximab, which targets CD20. (**c**) A glycoengineered antibody with a reduced fucose Fc region (purple), improving the antibody-dependent cellular cytotoxicity (ADCC). A notable example is obinutuzumab, which also targets CD20. (**d**) An antibody–drug conjugate with cytotoxic payload (red). A notable example is polatuzumab vedotin, which targets CD79b and delivers a payload of monomethyl auristatin E. (**e**) A bispecific antibody with distinct FAb complementarity. A notable example is glofitamab, which targets CD20 and CD3. Binding CD3 on a CD8+ T-cell activates its cytotoxic activity toward the approximated CD20+ cell.

Antibody therapies have revolutionized the landscape of lymphoid malignancy therapies since their inception. In 1975, Kohler and Milstein developed the first method for fusing murine B lymphocytes with immortal myeloma cells as a bioreactor to produce monoclonal antibodies with in vitro immortality. These fusion cell lines, or “hybridomas”, had promising clinical applications [1]. However, pure murine antibodies were limited in terms of their efficacy due to the short half-life, poor antibody-dependent cellular cytotoxicity (ADCC), and development of human anti-mouse antibodies (HAMA) in almost all patients with repeated infusion [2]. However, the core concept of developing targeted therapies that activate the patient’s own immune system was sound, particularly in the context of lymphoid malignancies, where a monoclonal species with unique targetable surface proteins is the source of disease. These therapies have been continually refined, from chimerized, to glycoengineered, to modern novel therapies, including antibody–drug conjugates (ADCs) and bispecific antibodies (BsAbs), as noted in Figure 1. Novel therapies are constantly being developed, and new combination regimens are now under investigation, with promising results. A summary of the reviewed studies can be found in Table 1.

## 2. Chimeric Monoclonal Therapies—Rituximab

### 2.1. Introduction

The first step beyond pure murine monoclonal therapies was the production of chimeric murine–human monoclonal antibodies. First described in 1984 by Morrison et al., these chimeric antibodies consisted of murine variable regions affixed to a human Fc region [43]. Utilizing DNA recombination techniques, murine hybridoma cells carrying genes for preselected variable regions are transfected with a viral vector carrying human Fc regions. The final product is a large (~150 dKa) IgG glycoprotein composed of a human Fc region with murine variable regions that have been selected for specific targets. The presence of a human Fc region improves the therapeutic potential of purely murine antibody therapies (-omabs) by improving the immunogenicity, half-life, and cell-mediated cytotoxic response [44,45]. Additionally, only ~10% of patients generate an anti-idiotype response of modest intensity to early chimeric antibodies [2].

Within the context of lymphoid malignancies, the most therapeutically notable chimeric antibody is rituximab, an anti-CD20 human–murine chimeric IgG monoclonal antibody [46]. CD20 is a transmembrane phosphoprotein that is initially expressed during early B-cell development and acts as a regulatory molecule in B-cell maturation. CD20 is expressed shortly before B-cells gain the ability to generate cytoplasmic heavy chains, with continued expression in mature B-cells until plasma cell differentiation. Thus, its high sensitivity and specificity to antibody-secreting B-cells makes CD20 an excellent target in B-cell malignancies.

**Figure 2:**
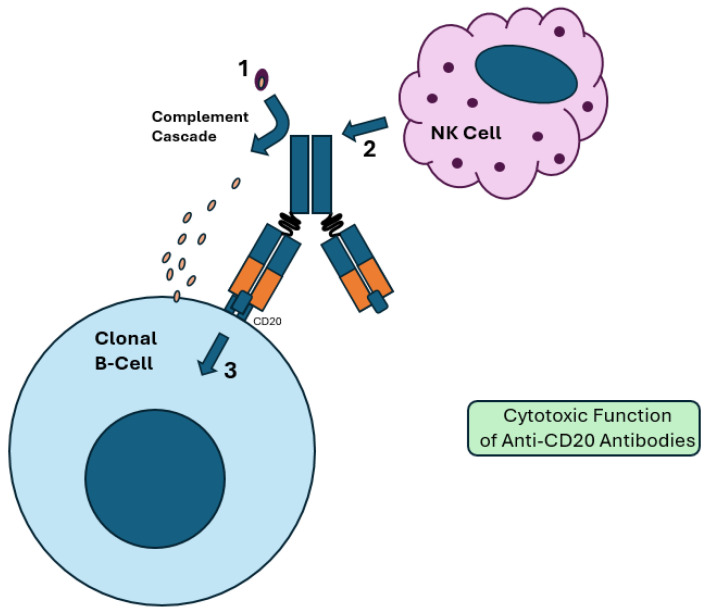
Cytotoxic function of anti-CD20 antibodies. (**1**) Proteolytic cleavage via the Fc region results in activation of the complement cascade. A membrane attack complex (MAC) is formed, resulting in complement-dependent cytotoxicity (CDC). (**2**) The Fc region of the bound antibody activates NK and other effector cells, resulting in antibody-dependent cellular cytotoxicity (ADCC). This is more effective in glycoengineered antibody therapies, which have a reduced fucose Fc region. (**3**) Binding of CD20 in clonal B-cells upregulates intracellular pathways that promote apoptosis. These include decreased expression of antiapoptotic proteins like BCL-2 and upregulated calcium influx, which results in cell death.

Once rituximab binds to CD20, the bound cells undergo cell death through a variety of mechanisms, which include complement-mediated cell death (CDC), ADCC, phagocytic activation, and multiple other cell death cascades, including upregulated calcium influx, as noted in Figure 2 [47,48]. This leads to the cytoreduction of CD20^+^ cell populations. Rituximab is then cleared by hepatic non-specific catabolism [49].

While rituximab has theoretical benefits in all CD20+ malignancies, it is best studied in chronic lymphocytic leukemia (CLL), non-Hodgkin lymphoma, including follicular lymphoma (FL), and diffuse large B-cell lymphoma (DLBCL) [50].

### 2.2. Clinical Applications

#### 2.2.1. Chronic Lymphocytic Leukemia

CLL is the most prevalent form of leukemia in the United States, accounting for 25% of diagnoses [51]. CLL is defined by the presence of a monoclonal B-cell population greater than 5 × 10^9^/L in the peripheral blood, with classic phenotypic characteristics of CLL, which include CD5+, CD19+, and CD43+ [3,4]. Rituximab monotherapy was a promising concept in CLL but has been largely inferior to combination therapies, with an overall response rate (ORR) of 51% and progression-free survival (PFS) of 18.6 months in previously untreated patients who were symptomatic or Rai stage 2–4 [5]. In patients who presented with Rai stage 0–2 disease, rituximab performed better, with an ORR of 81%, a median overall time to progression (TTP) of 23 months, and a time to retreatment (TTR) of 43 months (PFS was not assessed due to the high survival rates in this population) [6].

Rituximab was used throughout the 2000s in combination therapies, most notably the fludarabine and cyclophosphamide + rituximab regimen (FCR), which provided survival benefit as both new and salvage therapy [7].

While these combination regimens outperformed pure chemotherapy, they have been largely supplanted by the advent of targeted therapies. The MURANO trial was a phase III randomized study for patients with relapsed or refractory CLL that compared the efficacy of bendamustine + rituximab (BR) and venetoclax + rituximab (venR). Here, 62% of patients treated with venR achieved undetectable minimal residual disease (uMRD), while only 13% of patients in the BR arm reached uMRD, leading to higher rates of OS in the venR arm [8]. At the 5-year follow-up, the median PFS was 53.6 vs. 17.0 months, respectively, with 5-year OS of 82.1% vs. 62.2%, respectively [9].

Rituximab has also been shown to be highly efficacious in combination with targeted therapy. In the ECOG 1912 trial, patients with previously untreated CLL were treated with either FCR or ibrutinib + rituximab (IR). At three years, the IR arm outperformed FCR in PFS (89.4% vs. 72.9%, respectively), OS (98.8% vs. 91.5%, respectively) and tolerability, with decreased grade 3 or higher infectious complications in the IR arm [52,53].

#### 2.2.2. Follicular Lymphoma

FL is the third most common non-Hodgkin lymphoma, with an incidence of 17.1% in the United States [54]. It is classically characterized by a t(14;18), which results in overexpression of BCL-2, an antiapoptotic protein with resultant clonal expansion [3,10,11]. These clonal populations are nearly always positive for monoclonal immunoglobulin light chain, BCL-6, CD10, CD19, and CD20, making FL a prime target for anti-CD20 therapy [12].

Rituximab has been used for induction monotherapy in grades I–III follicular lymphoma with good effect. In a clinical trial by Colombat et al., 41% of the study population reached CR/CRu and 39% reached PR, with an ORR of 80% within the first year of therapy [13]. A study performed by the North Central Cancer Treatment Group also demonstrated an ORR of 72%, with only 56% of responders developing disease progression within 2.6 years [55]. The RESORT trial (E4402) tried to elucidate whether rituximab should be continued after induction. Patients were randomized to either maintenance rituximab (MR) or rituximab retreatment (RR), who only received treatment following disease progression. There was no statistical significance between the groups in terms of the primary endpoint of time to treatment failure (TTF). The MR group had an overall decreased dependency on cytotoxic therapy at three years and an improved duration of the first remission; however, the OS did not change at 10 years between the groups [14,15].

Rituximab’s first approval for FL was in 1997, following a phase II non-inferiority trial in relapsed/refractory FL as salvage therapy. Patients underwent 4 weekly infusions of rituximab. With an ORR of 48% and CR of 6%, rituximab performed similarly to single-agent cytotoxic regimens in this population [16]. Extending the therapy to eight weeks improved the outcomes further, with an ORR of 57%, of which 14% achieved CR [17].

This improved performance with longer therapy windows led to a landmark study evaluating the benefit of maintenance rituximab therapy. In the PRIMA trial, patients were separated into an observation group or given 2 years of maintenance rituximab monotherapy after induction with either CVP, CHOP, or FCM. At two years, 71.5% of patients in the rituximab group were in CR or uCR compared to 52.2% in the observation group [18]. Additionally, the PFS was 74.9% vs. 57.6%, respectively. Of note, the overall survival did not change between the groups after long-term follow-up analysis [19].

Rituximab in combination with chemotherapy has also been evaluated. The BRIGHT study evaluated bendamustine + rituximab (BR) against chemotherapy + rituximab regimens (R-CHOP or R-CVP) in untreated patients with indolent NHL or MCL. The differences in the complete response rate and OS were not statistically significant between the groups. However, the 5-year PFS was improved in the BR group at 65.5% compared to 55.8% in the R-CHOP / R-CVP group, and the adverse effect profile of the BR group was considered more favorable, with higher incidences of vomiting and infusion reactions but lower incidences of peripheral neuropathy and alopecia [56,57].

More recently, rituximab has been studied in combination with lenalidomide in the phase III multicenter AUGMENT trial for patients with relapsed or refractory indolent FL and MZL. Lenalidomide has shown increased NK-cell-mediated cytotoxicity and ADCC and so enhances the efficacy of rituximab. Patients were randomized to either a rituximab–placebo arm or a rituximab + lenalidomide. The lenalidomide group had a significantly long median PFS at 29.4% compared with the placebo at 14.1%. However, more adverse effects were noted in the lenalidomide group, most notable neutropenia (58% vs. 23%, respectively) [58].

#### 2.2.3. Diffuse Large B-Cell Lymphoma

DLBCL is the most common form of NHL, comprising nearly 30–40% of NHL diagnoses in the United States [59]. DLBCL is often aggressive and can be life-threatening without prompt initiation of treatment. These NHLs tend to express pan-B-cell markers, including CD19, CD20, CD22, CD45, and CD79a, making them a target for anti-CD20 therapies [60]. At a basic level, these malignancies can be separated into three distinct treatment groups: germinal-center B-cell type (GC), non-germinal center (non-GC), also known as activated B-cell type (ABC), and high-grade DLBCL, not otherwise specified [3]. Germinal-center DLBCL is characterized by CD10+ and is considered the lowest risk. High-grade DLBCL has an MYC gene rearrangement in combination with a BCL-2 or BCL-6 rearrangement, which confers the highest risk of a poor outcome. Non-GC DLBCL is considered an intermediate risk and is CD10- without rearrangement in the MYC plus BCL-2 or BCL-6 genes.

Rituximab as a monotherapy has not been shown to outperform the standard of care in DLBCL, including cutaneous forms [61,62]. In combination with cyclophosphamide, doxorubicin, vincristine and prednisone (CHOP), however, rituximab has demonstrated significant improvements in PFS and OS in low-risk young patients and high-risk elderly patients [20,21,63]. This regimen is widely used for initiation therapy in both GC and non-GC DLBCL. In high-grade DLBCL, rituximab in combination with DA-EPOCH (etoposide, prednisone, vincristine, cyclophosphamide, and doxorubicin) has conferred improved PFS rates at two years [64].

The combination with other regimens has also been studied. In young patients with high-risk disease, rituximab in combination with doxorubicin, cyclophosphamide, vindesine, bleomycin, and prednisone (R-ACVBP) outperforms R-CHOP, with improved 3-year EFS (81% to 67%) and OS (92% to 84%, respectively) [65]. The more recent POLARIX trial subgroup analyses demonstrated improved 2-year PFS in non-GC DLBCL patients who received Pola-R-CHP (polatuzumab vedotin, rituximab, cyclophosphamide, doxorubicin, prednisone) against a standard of care R-CHOP arm [66].

### 2.3. Adverse Effects of Rituximab

#### 2.3.1. Infusion-Related Reactions

In a 5-year review of infusion reactions to rituximab at a large academic infusion center, there was ~77% incidence of mild to severe infusion reactions, with 63% occurring during the first transfusion. Most of these reactions were classified as mild to moderate, with only 12% progressing to severe or life-threatening. Moreover, 84% of patients with grade 2 moderate reactions tolerated a same-day rechallenge [67].

#### 2.3.2. B-Cell Depletion and Immunosuppression

In a retrospective review, patients treated with rituximab for rheumatologic and lymphoid malignancies had a 73.3% chance of having an infection event (IE) within 3 months of infusion. In addition, 58% of the infections in the malignancy group were severe, requiring hospitalization [68]. These IEs often coincide with leukopenia, with a median onset time of 13 days following infusion, or late-onset neutropenia, which typically occurs at least four weeks following infusion [69]. Patients with chronic or latent hepatitis B have a 50–64% risk of reactivation following rituximab initiation [70]. Additionally, patients with NHL started on rituximab are at a 12.6% risk of reactivation of herpes zoster, with an odds ratio of 1.38 compared to the general population [71]. These adverse effects have been mitigated by recombinant VZV vaccination prior to treatment, hepatitis B screening prior to initiation, and close follow-up during initiation to screen for early signs of infection [72].

### 2.4. Resistance Mechanisms

Tumor cell lines have exhibited multiple resistance mechanisms to rituximab, including complement inhibition via upregulation of CD55 and CD59, internalization of CD20 complexes, and alteration of pro-apoptotic and anti-apoptotic pathways [73,74,75,76]. The most common resistance mechanism seen is decreased total CD20 surface expression, which is mediated by the MS4A1 gene. This effectively hides the tumor cell from rituximab and immune surveillance and is commonly found in relapsed/refractory DLBCL (R/R DLBCL) [76,77].

## 3. Glycoengineered Antibody Therapies—Obinutuzumab

### 3.1. Introduction

Obinutuzumab is a type II, glycoengineered monoclonal antibody that binds to CD-20, similarly to rituximab. However, obinutuzumab binds to a distinct epitope on the CD20 protein with a different orientation, allowing for a unique mechanism of action [78]. An IgG1 subclass, this glycoengineered antibody has a modified Fc region of reduced fucose content. This allows for enhanced ability to engage with the FcγRIII receptors of immune effector cells, like natural killer (NK) cells [22].

This antibody demonstrates several mechanisms that enhance its effectiveness in targeting CD20-expressing B-cells. First, it enhances ADCC through FcγRIII-mediated CD20 internalization, allowing for greater recruitment of immune effector cells, which increases the lysis of CD20-expressing B-cells. Additionally, it induces homotypic aggregation, where the clustering of B-cells results in a lysosome-mediated, non-apoptotic form of cell death, which is caspase-independent [78]. Furthermore, it promotes antibody-dependent cellular phagocytosis by enhancing the interactions with FcγRIII receptors, leading to greater recruitment and activation of macrophages, which then engulf the B-cells [79].

### 3.2. Clinical Applications

In the setting of lymphoid malignancies, obinutuzumab is FDA-approved for the treatment of CLL, FL, and MZL. In CLL, the drug is combined with chlorambucil in treatment-naive patients. A phase III trial showed that this combination (Clb-Obi) improved the median progression-free survival (26.7 months) compared to patients treated with rituximab plus chlorambucil (15.2 months) and chlorambucil alone (11.1 months). It also improved the overall survival, with a hazard ratio for death of 0.41 (*p* = 0.002) [80]. More recently, obinutuzumab in combination with venetoclax (Ven-Obi) was shown to significantly outperform Clb-Obi in terms of the median PFS (76.2 vs. 36.4 months), six-year rate of time to next treatment (65.2% vs. 37.1%), and rates of uMRD in bone marrow at three months (57% vs. 17%) [81].

In FL, the drug is combined with chemotherapy, followed as a monotherapy, in patients with treatment-naive stage II bulky, III, or IV FL. It can also be used in combination with bendamustine, followed by monotherapy, for patients with relapsed disease or disease refractory to a regimen containing rituximab [82].

Due to their similarities, obinutuzumab and rituximab have had their efficacy compared in several head-to-head trials. In the GALLIUM trial, patients with treatment-naive FL were randomized to the obinutuzumab + chemotherapy or rituximab + chemotherapy arm for first-line treatment. At 7 years, obinutuzumab outperformed rituximab, with a PFS of 63.4% and 55.7%, respectively [24,25]. In the GAUSS trial, 175 patients with relapsed indolent CD20+ DLBCL who responded to rituximab but developed progression were randomized to either a rituximab maintenance or obinutuzumab arm. The obinutuzumab arm had an independently confirmed improvement in the ORR (44.6% vs. 26.7%, respectively) without statistical improvement in the PFS [23]. However, the GOYA trial demonstrated that there was no statistical difference in PFS when compared to rituximab in combination with CHOP for first-line treatment of DLBCL, with an increased adverse event rate [27,83]. Thus, in summary, it does appear the obinutuzumab has improved efficacy for indolent lymphoid neoplasms; however, in aggressive lymphomas, there has not been demonstrated a significant clinical benefit over rituximab.

### 3.3. Adverse Effects of Obinutuzumab

However, the drug has some weaknesses. Infusion-related reactions (IRRs) are common, with the GALLIUM study reporting that 59.3% of patients receiving obinutuzumab experienced IRRs, compared to 48.9% of patients receiving rituximab (*p* < 0.001). These reactions included nausea, chills, fever and emesis, with more severe IRRs occurring in 12% of patients compared to 8% with rituximab. Careful monitoring and vigilance may be necessary with obinutuzumab, as well as premedication with Tylenol, antihistamines and corticosteroids to mitigate the severity of the IRRs [25]. Additionally, hematologic toxicities have been reported, with the GREEN trial indicating that 49.9% of patients developed neutropenia and 16.4% of patients developed thrombocytopenia when taking obinutuzumab [84].

## 4. Antibody–Drug Conjugate (ADC) Therapies

### 4.1. Introduction

Antibody–drug conjugate (ADC) therapies are an emerging class of targeted cancer treatments. Structurally, ADCs involve a tumor-specific monoclonal antibody (mAb), a cytotoxic molecule called the payload, and a covalent chemical bond that links the mAb and the payload. This design allows the targeted delivery of cytotoxins to cancerous cells while minimizing the off-target effects on normal tissue.

**Figure 3:**
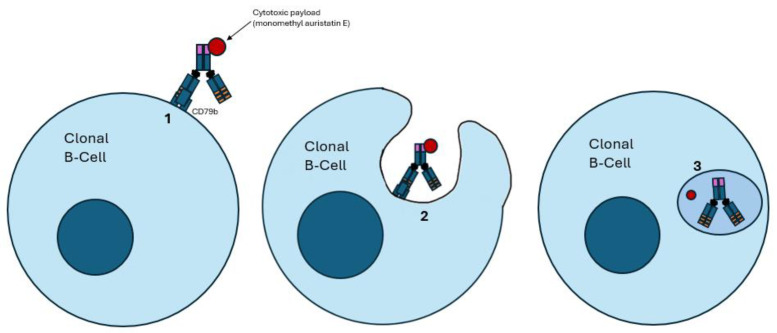
Mechanism of action of antibody–drug conjugates (ADCs). (**1**) The ADC binds to its target antigen; in this example, CD79b. (**2**) CD79b binding leads to endocytosis of the ADC. (**3**) The ADC undergoes proteolytic cleavage once it enters a lysosome, resulting in the freedom of the bound cytotoxic payload, which results in cell death. A notable example is polatuzumab vedotin, an anti-CD79b ADC, which carries a payload of monomethyl auristatin E.

Once ADCs bind to the corresponding antigen of tumor cells, the ADC–antigen complex is internalized. The cytotoxic payload is then released by the linker once the ADC undergoes lysosomal degradation, allowing the linker to bind to its intracellular target, resulting in cell death, as depicted in Figure 3. ADCs also exhibit “bystander killing” by diffusing into adjacent cells and inducing cell death. This design allows ADCs to specifically target tumor cells while reducing the damage to healthy tissue [28,29,85].

### 4.2. Clinical Applications

Clinically, ADCs have been particularly effective in treating lymphoid malignancies, where cell surface markers like CD30, CD22 and CD79b are commonly expressed on malignant lymphocytes. The FDA-approved ADCs for lymphoid malignancies include loncastuximab teserine, brentuximab vedotin, inotuzumab ozogamicin, moxetumomab pasudotox, and polatuzumab vedotin.

Loncastuximab teserine (LT) is an ADC that targets the B-cell marker CD19. Its payload, SG3199, is an irreversible DNA cross-linker that leads to cell death. The LOTIS-2 trial demonstrated efficacy in R/R DLBCL, with a CR rate of 24.8%. While the median PFS was 4.9 months and the median OS was 9.5 months, patients who achieved CR surpassed these values, with a 2-year PFS rate of 72.5% and 2-year OS rate of 68.2% [86,87]. The LOTIS-3 trial exploring the use of LT in combination with ibrutinib for R/R DLBCL had promising preliminary data, with an ORR of 57.1% and overall CRR of 34.3%, but was terminated early. The LOTIS-5 trial investigating a regimen of LT with rituximab is ongoing, with recruitment scheduled until 2028 and with preliminary data showing an ORR of 80% and a CRR of 50% [26].

Brentuximab vedotin (BV) targets CD30, a TNF receptor found on Reed–Sternberg cells in Hodgkin lymphoma, anaplastic large-cell lymphoma cells, and some cutaneous T-cell lymphoma cells. The cytotoxic component of BV is monomethyl auristatin E, a microtubule-inhibiting agent [88]. In the recently updated ESCHELON-1 trial, BV in combination with doxorubicin, vinblastine, and dacarbazine (A+AVD) was placed head to head against the gold standard therapy of doxorubicin, bleomycin, vinblastine, and dacarbazine (ABVD) for first-line treatment in patients with untreated stage III or IV classical Hodgkin lymphoma. A+AVD outperformed ABVD in seven-year OS (94.6% vs. 89.4%, respectively), with maintained PFS rates of 82.3% vs. 75.3%. A+AVD also had a favorable AE profile, with the removal of bleomycin decreasing the pulmonary adverse outcomes [89,90,91]. A phase II study in patients with relapsed or refractory Hodgkin lymphoma following autologous stem-cell transplantation has also shown a 75% overall response rate, with complete remission in 34% of patients [92].

Polatuzumab vedotin targets CD79b, a component of the B-cell receptor complex. This ADC has shown significant clinical efficacy in treating DLBCL. Similarly to BV, its cytotoxic payload is monomethyl auristatin E. In the phase II GO29365 study, the combination of polatuzumab vedotin with bendamustine and rituximab significantly improved the complete response rate (40% vs. 17.5%, *p* = 0.026) and progression-free survival (median of 9.5 months vs. 3.7 months, *p* < 0.001, HR 0.36) compared to bendamustine and rituximab alone [93]. The POLARIX trial demonstrated the efficacy of polatuzumab vedotin with rituximab, cyclophosphamide, doxorubicin, and prednisone (Pola-R-CHP) against the standard-of-care R-CHOP. The two-year PFS in Pola-R-CHP was 76.7% compared to 70.2% for R-CHOP [30]. A subgroup network meta-analysis revealed improved hazard ratios in patients with untreated activated B-cell-like (ABC) DLBCL, an aggressive and generally treatment-resistant subtype, who were treated with Pola-R-CHP when compared to other regimens, including R-CHOP plus bortezomib (HR: 0.52, *p* = 0.02), R-CHOP plus ibrutinib (HR: 0.43, *p* = 0.001), R-CHOP plus lenalidomide (HR: 0.51, *p* = 0.009), obinutuzumab-CHOP (HR: 0.46, *p* = 0.008), and R-CHOP (HR: 0.40, *p* < 0.001) [31].

### 4.3. Adverse Effects of ADCs

While effective, ADCs have a notable AE profile and several described resistance mechanisms. Since tumor antigens are not specific to tumor cells, toxicity can theoretically occur to all mitotically active cells when a potent cytotoxic is delivered by an ADC. For example, toxicity, including grade 3–4 neutropenia, anemia, thrombocytopenia and peripheral neuropathy are clear with polatuzumab vedotin [85]. The resistance mechanisms also limit the clinical efficacy of ADCs through tumor-induced downregulation or mutation of the target antigen, resulting in suboptimal binding by the ADC. Furthermore, there have been cases of multidrug resistance 1 (MDR1) gene overexpression, with consequent P-glycoprotein-mediated drug efflux of the cytotoxic payload [33,94]. However, ongoing research aims to overcome these challenges. Efforts include developing novel linkers to enhance the bystander effect and designing unique antibody structures to stabilize the therapeutic potential of ADCs.

## 5. Bispecific Antibodies—Immunotherapy

### 5.1. Introduction

**Figure 4:**
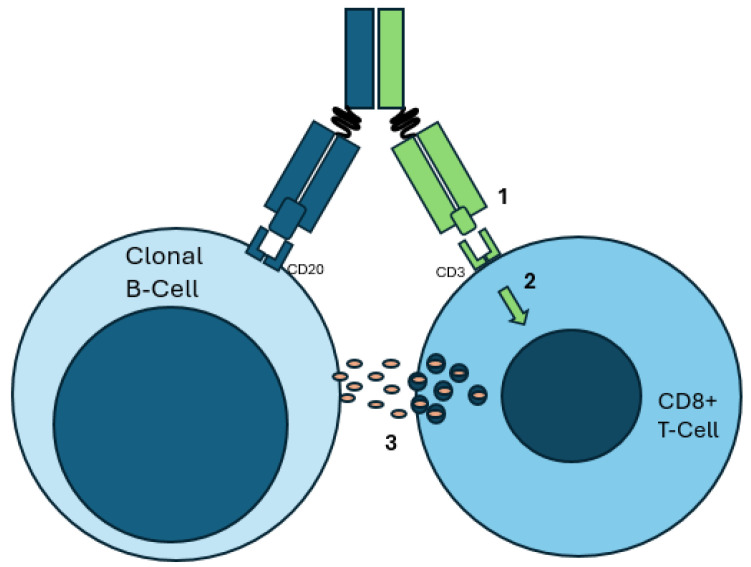
Example of a malignant B-cell as targeted by a CD20xCD3 bispecific antibody therapy. Note the approximation of a CD3+ T-cell with the clonal B-cell via the bispecific antibody. (**1**) The bispecific antibody binds to CD20 on the clonal B-cell and CD3 on the CD8+ T-cell. (**2**) CD3 binding leads to TCR-independent signaling, resulting in activation. (**3**) Cytotoxic granules and cytokines are released, resulting in immune activation and clonal B-cell demise.

Bispecific antibodies (BsAbs) offer another innovative therapeutic approach. Structurally, they are designed to bind two unique antigens simultaneously, often redirecting immune effector T-cells to the tumor cell, to induce cytotoxicity [95]. BsAbs can be divided into two main categories, IgG-like and non-IgG-like BsAbs. IgG-like BsAbs resemble natural antibodies and include an Fc region, which stabilizes and extends the half-life of the drug and enables immune effector processes, including antibody-dependent cellular cytotoxicity and complement-dependent cytotoxicity. However, IgG-like antibodies can be challenging to engineer due to potential mispairing of the heavy and light chains. In contrast, non-IgG-like BsAbs lack an Fc region, making them smaller and with a shorter half-life. However, without the need for Fc receptor interactions, they have a more flexible and straightforward design. Bispecific T-cell engagers (BiTEs) are a type of non-IgG-like BsAb [34]. Functionally, BsAbs facilitate the recruitment and activation of T-cells, resulting in tumor cell lysis, as seen in Figure 4. One arm targets a tumor cell antigen, such as CD19, CD20 or BCMA, while the other arm binds to the CD3 antigen on T-cells. This CD3 engagement on cytotoxic CD8+ T-cells upregulates activation markers and releases inflammatory cytokines, driving tumor cell lysis. The dual binding function of BiTEs forms an immunologic synapse between the T-cell and the tumor cell, triggering T-cell activation, proliferation and tumor cell destruction. [34,95]. Given their mechanism of action, BsAbs have a unique side effect profile, including CRS and neurological toxicity related to their ability to stimulate the immune system [32].

### 5.2. Clinical Applications

Clinically, BsAbs have shown great promise. Blinatumomab, a BiTE that targets CD19 and CD3, is particularly effective in treating B-cell acute lymphoblastic leukemia, especially in patients with minimal residual disease (MRD). The phase II BLAST study demonstrated that 78% of patients with the B-cell precursor ALL in complete remission but with MRD achieved a great MRD response after one cycle of blinatumomab [35]. Similarly, mosunetuzumab, a BsAb that targets CD20 and CD3, has shown efficacy in treating relapsed or refractory B-cell non-Hodgkin lymphoma. A phase I/II clinical trial demonstrated that mosunetuzumab achieved an ORR of 66% and a CRR of 48.5% in patients with indolent relapsed or refractory B-cell non-Hodgkin lymphoma [96].

More recently, there have been multiple CD3xCD20 BsAbs with approval as third-line therapies in lymphoid malignancies. Epcoritamab is a subcutaneous CD3xCD20 BsAb, which recently obtained FDA approval for third-line treatment of R/R DLBCL. In the EPCORE NHL-1 trial, patients with R/R DLBCL treated with epcoritamab had a 24-month follow-up ORR of 63.1%, with 40.1% achieving CR. The estimated 24-month PFS was 65.1%, with an OSR of 78.2%. Patients with R/R FL were also evaluated, with an ORR of 82% and a CR of 62.5%, although FDA approval for third-line therapy has not been granted [36,37,97]. Glofitamab, another promising CD3xCD20 BsAb, has also received accelerated approval for third-line treatment of R/R DLBCL and LBCL arising from FL. These patients are pretreated with obinutuzumab to mitigate the risk of cytokine release syndrome (obinutuzumab depletes the number of B-cells available as a target). Patients achieved a CRR of 39% at 12.6 months. Of note, the response was independent of prior treatment, including CAR-T and histological, or molecular subtype apart from the high-grade DLBCL cohort, which only experienced PR [38]. Finally, mosunetuzumab has also received approval for the treatment of R/R FL. In the clinical trial GO29781, patients with R/R FL who had failed two prior therapies achieved an ORR of 80%, and a CRR of 60%, with median PFS of 17.9 months. The current regimen uses step-up dosing to mitigate the main adverse effect of CRS [39,96].

### 5.3. Adverse Effects of Bispecific Antibody Therapies

Given their mechanism of action, the AE profile of BsAbs is generally characterized by cytokine release syndrome (up to 72% in LBCL), neutropenia with hypogammaglobulinemia, CNS toxicity, and infections, which are primarily driven by T-cell activation [32].

## 6. Future Directions

### 6.1. Combination Regimens

There is active investigation into the use of antibody therapy for patients with lymphoid malignancies. Fortunately, antibodies are readily able to be administered with one another to allow for combination therapy, thus increasing the potential of synergy. To date, the most common tumor-associated antigen targeted has been CD20; however, ongoing investigation into other targets is actively being pursued. However, we do not expect CD20-directed therapies to be abandoned in the near future. Unlike cellular therapies, there is a lot more potential for novel treatment approaches with antibodies. From a clinical perspective, ongoing studies are investigating the use of BsAbs and ADCs in combination with each other and with chemotherapy regimens like R-CHOP as early-line therapies in both DLBCL and FL.

The EPCORE NHL-2 trial is underway to evaluate the combination of epcoritamab with other regimens. Preliminary data from several arms are promising. Arm 1 is actively investigating epcoritamab in combination with R-CHOP for previously untreated DLBCL. Phase 1 and 2 early data report an ORR of 100% and a complete metabolic response (CMR) of 96% at 9 months. Notably, the response rate was similar across all the DLBCL subtypes [98]. They have recently begun a phase III trial (NCT04663347).

Arm 6 of the study is investigating the use of epcoritamab in combination with lenalidomide for previously untreated FL. Early data presented in abstract show an ORR of 95% and a CRR of 85% at the 21-month follow-up. Maintenance therapy with epcoritamab in patients with FL who have undergone standard-of-care treatment is being investigated in arm 7, where all eight patients enrolled with PR have had a 100% conversion rate to CR, with an acceptable AE profile thus far [99]. These findings are still preliminary but are hopeful.

Arm 9 of EPCORE NHL-2 is still in the recruitment phase and is set to investigate epcoritamab + lenalidomide for second-line treatment in patients with R/R FL who progressed within 24 months of initiation of first-line anti-CD20-containing immunochemotherapy (NCT04663347).

BsAbs are also being investigated in combination with ADCs and immunotherapies. An ongoing phase 1b/2 trial of mosunetuzumab in combination with polatuzumab for R/R LBCL has reported an ORR and a CRR of 59.2% and 45.9%, respectively, at a median of 23.9 months. Notably, the rate of CRS was only 16.7% in the early data [100]. A recent subgroup update of the phase 1b/2 trial reported no statistical difference in the durable response regardless of prior treatment history, with a CR of 35.9% (95% CI 21.9–51.2) in the refractory group and a CRR of 59.3% (95% CI 38.8–77.6) in the relapsed group [101]. Combination therapy of glofitamab and polatuzumab is also under investigation in R/R DLBCL [102]. Epcoritamab and Pola-R-CHP are being investigated as first-line therapy in patients with DLBCL in the phase 1b/2 EPCORE NHL-5 study, with an early reported ORR of 100% and CRR of 89% at a median time to follow-up of 5.8 months [103]. Finally, preliminary results from a phase 1b/2 trial for mosunetuzumab in combination with lenalidomide in patients with previously untreated FL reported an ORR of 88.9% and a CR of 81.5% at the time of reporting. No median follow-up was reported (NCT04246086) [104]. These results promoted a phase III trial, which is ongoing (NCT04712097).

CD47 is another novel target that has been identified in recent years, with several exciting developments. CD47, the so-called “Don’t Eat Me” signal, is a surface protein that is expressed in normal cells as an inhibitor of phagocytosis by macrophages and dendritic cells. CD47 binds the SIRPα receptor on these phagocytic immune surveillance cells and prevents phagocytosis. CD47 is commonly highly expressed in tumor cells. Several therapeutic antibody therapies are being developed to block this pathway with the goal of increasing immune surveillance and the death of tumor cells [105].

The first described anti-CD47 therapy is magrolimab, a humanized anti-CD47 antibody. Magrolimab has been primarily studied in combination therapy with CD20 (NCT02953509]. In R/R NHL, patients treated with magrolimab + rituximab achieved an ORR of 52.2% and a CR of 30.4%. The median PFS at 3 years was 7.4 months, with 21% having developed grade III/IV anemia [40,41]. In R/R DLBCL, magrolimab + rituximab (MR) was compared to a regimen of MR, gemcitabine, and oxaliplatin (MR+GemOx). The regimens achieved ORRs of 24.2% and 51.5%, with CRRs of 12.1% and 39.4%, respectively. Notably, there was one treatment-related death (grade V colitis) in the MR+GemOx arm [42]. Ligufalimab, another anti-CD47 humanized antibody therapy, is currently under investigation for multiple malignancies (NCT0472830) [106].

There are many other therapies targeting the CD47 pathway in the pipeline, with 15 current clinical trials [105]. A notable example is IMM0306, a BsAb targeting CD20 and the CD47-binding domain of SIRPα [107]. IMM0306 is being studied both as a monotherapy for R/R CD20+ B-cell NHL and in combination with lenalidomide (NCT05805943 and NCT05805943, respectively). There are not yet preliminary data from these studies.

### 6.2. Role of Machine Learning

From a therapy development perspective, antibody therapies should continue to address the common shared weaknesses while improving clinical efficacy by improving target selection, immunogenicity, poor tumor penetration/resistance mechanisms, and difficulties with production or delivery (e.g., high production cost, low half-life).

The problems of immunogenicity and poor tumor penetration are both related to the core structures that form the therapeutic antibody. Researchers are addressing these problems with predictive modeling via machine learning algorithms. Identifying and avoiding peptide motifs associated with high anti-therapy immunogenicity will theoretically decrease adverse effects and improve efficacy. Machine learning models offer the ability to predict the possible anti-therapy immunogenicity of future antibody therapies. Currently, there are many different models that aim to improve the drug development pipeline at multiple stages, including analyzing tumor genomics, identifying targetable motifs, and predicting immunogenicity [108]. In 2020, Liang and Zhang generated a machine learning model that predicted immunogenicity with 83% accuracy when given crystalized structures of therapeutic monoclonal antibodies, and 65% accuracy with modeled structures during leave-one-out cross-validation [109]. Another example is TAP 1.0, an ML system designed to analyze protein sequences to identify potential tumor antigens with high immunogenicity for future targeted therapies. The use of such processes has the potential to greatly decrease the development cost of targeted therapies by decreasing broad in vitro assays, which are both time- and labor-intensive [110]. These advancements show great promise in improving the feasibility and development of targeted therapies for lymphoid malignancies.

## Figures and Tables

**Table 1:** Summary of antibody therapies in lymphoid malignancies, organized by study, population, therapy regimen with target, and primary outcomes listed, with *p*-values included where available.

Citations	Study Population	Studied Therapy	Therapy Target	Primary Outcomes
**Chimeric** **Therapies—Rituximab**				
Hainsworth et al., 2003 [3]	Untreated Rai stage II–IV or Rai stage I symptomatic CLL	Rituximab induction monotherapy vs. standard-of-care induction chemotherapy	CD20	ORR 51%; average PFS 18.6 months
Ferrajoli et al., 2011 [4]	Untreated Rai stage 0–2 CLL	Rituximab induction monotherapy vs. induction cyclophosphamide, vincristine, and prednisone (CVP)	CD20	ORR 81%; TTP 23 months; TTR 43 months
Hallek et al., 2010 [5]	Untreated CLL	Fludarabine + cyclophosphamide + rituximab (FCR) vs. bendamustine + rituximab (BR)	CD20	3-year PFS rate 65% vs. 45% in control group (*p* < 0.0001)
MURANO trial with 5-year follow-up (Seymour et al., 2018, 2022) [6,7]	Relapsed/refractory CLL	Venetoclax + rituximab (venR) vs. bendamustine + rituximab (BR)	CD20	uMRD rate 62%; 5-year median PFS 53.5 months (*p* < 0.001); 5-year OS rate 82.1% (*p* < 0.001)
ECOG 1912 trial with 3-year follow-up (Shanafelt, 2019, 2022) [8,9]	Previously untreated CLL	Ibrutinib + rituximab (IR) vs. fludarabine, cyclophosphamide, and rituximab (FCR)	CD20	5.8-year PFS rate 89.4% (*p* < 0.001); 3-year OS rate 98.8% (*p* < 0.001)
Colombat et al., 2001 [10]	Untreated grade I–III FL	Rituximab induction monotherapy	CD20	1-year ORR 80%, 1-year CRR 41%
Witzig et al., 2005 [11]	Untreated stage III/IV FL	Rituximab induction monotherapy	CD20	ORR 72% (*p* = 0.02); 2.6-year PFS 46%
RESORT trial with 10-year follow-up (Kahl et al., 2014, 2024) [12,13]	Untreated low-burden FL	Maintenance (MR) vs. retreatment (RR) rituximab	CD20	No statistical difference in 10-year TTF, OS
Piro et al., 1999 [14]	Relapsed/refractory NHL	Rituximab monotherapy vs. historical group that did not receive rituximab	CD20	ORR 57%; CRR 14%
PRIMA trial with 9-year follow-up (Salles et al., 2011, Bachy et al., 2019) [15,16]	Untreated FL	Maintenance rituximab after immunochemotherapy	CD20	9-year PFS rate 51.1% vs. 35.0% in control group (*p* = < 0.001); no statistical difference in OS
BRIGHT study with 5-year follow-up (Flinn et al., 2014, 2019) [17,18]	Untreated, indolent NHL or MCL	Bendamustine + rituximab (BR) vs. R-CHOP or R-CVP	CD20	5-year PFS 65.5% in BR group vs. 55.8% in R-CHOP/R-CVP (*p* = 0.0025)
AUGMENT trial (Leonard et al., 2019) [19]	Relapsed/refractory FL or marginal zone lymphoma	Lenalidomide + rituximab vs. placebo + rituximab	CD20	PFS 39.4 months for lenalidomide + rituximab vs. PFS 14.1 months for placebo + rituximab (*p* < 0.001)
Oki et al., 2014 [20]	Double-hit lymphoma	R-CHOP, R-EPOCH, and R-hyperCVAD/MA	CD20	2-year event-free survival: 33% R-CHOP, 25% R-EPOCH, 67% hyperCVAD/MA
Récher et al., 2011 [21]	Untreated DLBCL	Rituximab + doxorubicin, cyclophosphamide, vindesine, bleomycin and prednisone (R-ACVBP) vs. R-ECHOP	CD20	3-year event free survival 81% R-ACVBP vs. 67% R-CHOP (*p* = 0.0035)
**Glycoengineered Therapies—Obinutuzumab**				
Al-Sawaf et al., 2024 [22]	Untreated CLL	Venetoclax–obinutuzumab (Ven-Obi) or chlorambucil-obinutuzumab (Clb-Obi)	CD20	PFS 76.4 months for Ven-Obi vs. 36.4 months for Clb-Obi
GAUSS trial (Sehn et al., 2015) [23]	Relapsed indolent DLBCL	Obinutuzumab vs. rituximab	CD20	ORR 44.6% obinutuzumab vs. 26.7% rituximab (*p* = 0.01); no statistical difference in PFS
GALLIUM trial (Marcus et al., 2017; Townsend, 2023) [24,25]	Untreated FL	Obinutuzumab vs. rituximab	CD20	7-year PFS 63.4% obinutuzumab vs. 55.7% rituximab (*p* = 0.006), grade III/IV AE rate 4.5% vs. 4.4% respectively (*p* = 0.03)
**Antibody–Drug Conjugate Therapies**				
POLARIX trial (Tilly et al., 2021) [26]	Untreated DLBCL	Polatuzumab vedotin, rituximab, cyclophosphamide, doxorubicin, and prednisone (Pola-R-CHP) vs. R-CHOP	CD79b	2-year PFS 76.7% in Pola-R-CHP group vs. 70.2% in R-CHOP group (*p* = 0.02)
LOTIS-2 trial with 2-year follow-up (Caimi, 2021, 2024) [23,27]	Relapsed/refractory DLBCL	Loncastuximab tesirine (loncastuximab tesirine-lpyl [Lonca])	CD19	2-year PFS 72.5%, 2-year OS rate 68.2%
ESCHELON-1 trial with 6-year follow-up (Connors et al., 2018; Ansell et al., 2022) [28,29]	Untreated stage III/IV CHL	Brentuximab vedotin (BV) with doxorubicin, vinblastine, and dacarbazine (A+AVD) vs. doxorubicin, bleomycin, vinblastine, and dacarbazine (ABVD)	CD30	6-Year OS 94.6% A+AVD vs. 89.4% ABVD (*p* = 0.009)
**Bispecific Antibody Therapies**				
BLAST study (Goekbuget et al., 2014) [30]	B-cell precursor ALL	Blinatumomab monotherapy	CD19 and CD3	1-cycle MRD rate 78%
Budde et al., 2022 [31]	Relapsed/refractory B-cell non-Hodgkin lymphoma	Mosunetuzumab monotherapy	CD20 and CD3	3.5-year ORR 66.2%, 48.5% in indolent R/R NHL
Budde et al., 2022 [32]	Relapsed/refractory follicular lymphoma	Mosunetuzumab monotherapy	CD20 and CD3	IRC assessed post-treatment CRR 60% (*p* < 0.0001)
EPCORE NHL-1 trial (Linton et al., 2024) [33]	Relapsed/refractory DLBCL	Epcoritamab monotherapy	CD20 and CD3	2-year ORR 63.1%, with 40.1% achieving CR; PFS 65.1% with OSR of 78.2%
Dickinson et al., 2022 [34]	Relapsed/refractory DLBCL	Glofitamab monotherapy	CD20 and CD3	CRR of 39% at 12.6 months
**Combination Therapies**				
EPCORE NHL-2 trial, arm 1 (Clausen et al., 2023 *) [35]	Untreated high-risk DLBCL	Epcoritamab + R-CHOP	CD20 and CD3	Post-treatment CMR rate 76%, including double/triple-hit DLBCL; 9-month durable CMR rate 96%
EPCORE NHL-2 trial, arms 6+7 (Lori et al., 2024 *)	Untreated FL	Arm 6: induction epcoritamab + rituximab + lenalidomide (R^2); arm 7: maintenance epcoritamab + R^2	CD20 and CD3	Arm 6: 21-month ORR 95%, CRR 85%; arm 7: 19.7-month 83% sustained conversion rate to CR
Assouline et al., 2024 * [36]	Relapsed/refractory DLBCL	Mosunetuzumab + polatuzumab vedotin	CD20 and CD3; CD79a	IRC-assessed 33-month ORR 59.2%, CRR 45.9%
Hutchings et al., 2023 * [37]	Relapsed/ refractory DLBCL	Glofitamab + polatuzumab vedotin	CD20 and CD3; CD79a	Post-treatment ORR 78%, CMR 56%
EPCORE NHL-5 trial (Lavie et al., 2024) * [38]	Untreated DLBCL	Epcoritamab + Pola-R-CHP	CD20 and CD3	5.8-month ORR 100%, CRR 89%
Morschhauser et al., 2023 * [39]	Untreated FL	Mosunetuzumab + lenalidomide (M + Len)	CD20 and CD3	Post-treatment ORR 88.9%, CMR 81.5%
**Anti-CD47 Antibody Therapies**				
Sikic et al., 2019; Mehta et al., 2024 [40,41]	Indolent relapsed/refractory NHL	Magrolimab + rituximab (MR)	CD47, CD20	ORR 52.2%, CRR 30.4%, grade 3+ AE 71.7%
Maakaron et al., 2024 [42]	Relapsed/refractory DLBCL	Magrolimab + rituximab (MR) vs. MR, gemcitabine, oxaliplatin (MRGemOx)	CD47, CD20	MR: ORR 24.2%, CRR 12.1%, serious AE 46.5%; MRGemOx: ORR 51.5%, CRR 39.4%, serious AE 78.8%

* Early data from abstracts presented at major conferences. Abbreviations used: CLL: chronic lymphocytic leukemia; FL: follicular lymphoma; NHL: non-Hodgkin lymphoma; MCL: mantle cell lymphoma; DLBCL: diffuse large B-cell lymphoma; CHL: classical Hodgkin lymphoma; ORR: overall response rate; PFS: progression-free survival; TTP: time to progression; TTR: time to relapse; uMRD: undetectable minimal residual disease; OS: overall survival; CRR: complete response rate; TTF: time to failure; OSR: overall survival rate; CMR: complete molecular remission; AE: adverse event.
